# Linker-Improved Chimeric Endolysin Selectively Kills Staphylococcus aureus *In Vitro*, on Reconstituted Human Epidermis, and in a Murine Model of Skin Infection

**DOI:** 10.1128/aac.02273-21

**Published:** 2022-04-13

**Authors:** Fritz Eichenseher, Bjorn L. Herpers, Paul Badoux, Juan M. Leyva-Castillo, Raif S. Geha, Mathijs van der Zwart, James McKellar, Ferd Janssen, Bob de Rooij, Lavanja Selvakumar, Christian Röhrig, Johan Frieling, Mark Offerhaus, Martin J. Loessner, Mathias Schmelcher

**Affiliations:** a Institute of Food, Nutrition and Health, ETH Zurich, Zurich, Switzerland; b Micreos GmbH, Wädenswil, Switzerland; c Regional Public Health Laboratory Kennemerland, Haarlem, The Netherlands; d Boston Children’s Hospital, Harvard Medical School, Boston, Massachusetts, USA; e Micreos Human Health B.V., Bilthoven, The Netherlands

**Keywords:** atopic dermatitis, bacteriophage, endolysin, antibiotic resistance, microbiome

## Abstract

Staphylococcus aureus causes a broad spectrum of diseases in humans and animals. It is frequently associated with inflammatory skin disorders such as atopic dermatitis, where it aggravates symptoms. Treatment of S. aureus-associated skin infections with antibiotics is discouraged due to their broad-range deleterious effect on healthy skin microbiota and their ability to promote the development of resistance. Thus, novel S. aureus-specific antibacterial agents are desirable. We constructed two chimeric cell wall-lytic enzymes, Staphefekt SA.100 and XZ.700, which are composed of functional domains from the bacteriophage endolysin Ply2638 and the bacteriocin lysostaphin. Both enzymes specifically killed S. aureus and were inactive against commensal skin bacteria such as Staphylococcus epidermidis, with XZ.700 proving more active than SA.100 in multiple *in vitro* activity assays. When surface-attached mixed staphylococcal cultures were exposed to XZ.700 in a simplified microbiome model, the enzyme selectively removed S. aureus and retained S. epidermidis. Furthermore, XZ.700 did not induce resistance in S. aureus during repeated rounds of exposure to sublethal concentrations. Finally, we demonstrated that XZ.700 formulated as a cream is effective at killing S. aureus on reconstituted human epidermis and that an XZ.700-containing gel significantly reduces bacterial numbers compared to an untreated control in a mouse model of S. aureus-induced skin infection.

## INTRODUCTION

Staphylococcus aureus is an important Gram-positive opportunistic pathogen, which causes mortality and morbidity in humans and animals worldwide. While it colonizes 20 to 30% of healthy individuals, it can cause a wide spectrum of (often hospital-acquired) diseases in susceptible patients, ranging from relatively mild to life-threatening conditions (1, 2). This includes skin infections (such as impetigo, folliculitis, and cellulitis), wound and soft tissue infections, abscesses on skin and internal organs, osteomyelitis, pneumonia, meningitis, endocarditis and bloodstream infections, and sepsis (2, 3). S. aureus infections are often difficult to treat by conventional antibiotic therapy because the bacteria can hide in protected niches within the body, such as biofilms, abscesses, and intracellular compartments, where they frequently exist in a dormant state (4–6). Moreover, S. aureus readily acquires resistance to multiple antibiotics, with methicillin-resistant S. aureus (MRSA) being the most prominent example (7).

Atopic dermatitis (AD) is a chronic inflammatory skin condition that affects up to 25% of children and up to 10% of adults worldwide (8) and is associated with dry and itchy skin, which can have a significant impact on quality of life (9). S. aureus colonization and a reduced diversity of the skin microbiome are highly prevalent in AD patients, with 30% to 100% of these patients being colonized with S. aureus, depending on the method used (10). S. aureus can aggravate inflammation via secretion of virulence factors such as enterotoxins, which, in turn, trigger the release of proinflammatory cytokines (11). Vice versa, a weakened skin barrier as a frequent consequence of AD facilitates S. aureus colonization. AD is commonly treated with emollients, anti-inflammatory agents such as topical corticosteroids, immunosuppressants, or monoclonal antibodies, whereas antibiotic therapy aiming at the elimination of S. aureus is indicated only in clinically infected cases, for which AD patients have an increased risk (11–13). Treating S. aureus in AD with (broad-spectrum) antibiotics is discouraged due to their undesired impact on the healthy skin microbiome and the associated risk of antibiotic resistance induction (14). Instead, novel antimicrobial agents that specifically target S. aureus but are ineffective against coagulase-negative staphylococcal (CoNS) species such as Staphylococcus epidermidis and other beneficial commensal skin bacteria would be desirable.

Endolysins are bacteriolytic enzymes encoded by bacteriophages, which degrade from within the peptidoglycan (PG) bacterial cell wall at the end of the phage replication cycle, causing cell death and the release of progeny phages (15). In Gram-positive organisms, the PG is exposed to the outside due to the absence of an outer membrane, for which reason endolysins are effective against these bacteria also when added externally as recombinant proteins and have therefore been suggested as a novel class of antibacterial agents (16). The most important advantages of endolysins as antimicrobials are (i) their rapid killing kinetics (17), (ii) the high level of specificity for their target cells (18), (iii) their activity against drug-resistant bacteria, dormant cells, and biofilms (19, 20), and (iv) their low chance of inducing bacterial resistance (21) due to their highly conserved target sites in the PG. Staphylococcal phage endolysins have been shown to effectively kill staphylococci, both *in vitro* and in animal infection models (22). However, they are usually specific for staphylococci at the genus level (including S. aureus and CoNS) (20), whereas endolysins active exclusively against S. aureus have not been described. Staphylococcal endolysins feature a modular architecture, typically comprising two enzymatically active domains (EADs) and one SH3b-type cell wall binding domain (CBD). In most cases, the two EADs include an N-terminal cysteine-histidine-dependent amidohydrolase/peptidase (CHAP) domain cleaving the d-Ala-Gly bond of staphylococcal PG and one amidase domain targeting the MurNAc-l-Ala bond. In a “lysis from without” setting, the CHAP domains contribute most to the lytic activity of these enzymes, whereas the amidase domains are essentially inactive (23–25). The endolysin of staphylococcal phage 2638A (Ply2638) is different in the way that it features a highly active amidase domain and an N-terminal M23 endopeptidase instead of a CHAP domain, which targets the same PG bond (d-Ala-Gly) but contributes only little to the activity of the enzyme (20, 26).

Here, we describe the construction and functional characterization of two chimeric peptidoglycan hydrolases (PGHs) named Staphefekt SA.100 (SA.100) and XZ.700, which were generated to render the enzyme more specific toward S. aureus. This was accomplished by replacing the native low-activity M23 domain of Ply2638 by that of lysostaphin (LST), a bacteriocin produced by Staphylococcus simulans and directed against S. aureus (27). The M23 endopeptidase domain of LST targets the pentaglycine bridge (Gly-Gly activity), an interpeptide bridge unique to S. aureus PG, whereas CoNS feature modifications in their interpeptide bridges, which render them less susceptible to the action of LST (28, 29). SA.100 and XZ.700, while featuring a similar domain architecture, differ in a linker region connecting their M23 and amidase domains. We first compared both chimeric enzymes in multiple *in vitro* activity assays. Then, we further characterized the more active construct, XZ.700, investigating its specificity toward S. aureus, its potential to induce bacterial resistance, and its efficacy at killing S. aureus on reconstituted human epidermis (RHE) and in a mouse model of superficial skin infection.

## RESULTS

### Substitution of the M23 domain of the Ply2638 endolysin yields an active chimeric PGH.

**FIG 1:**
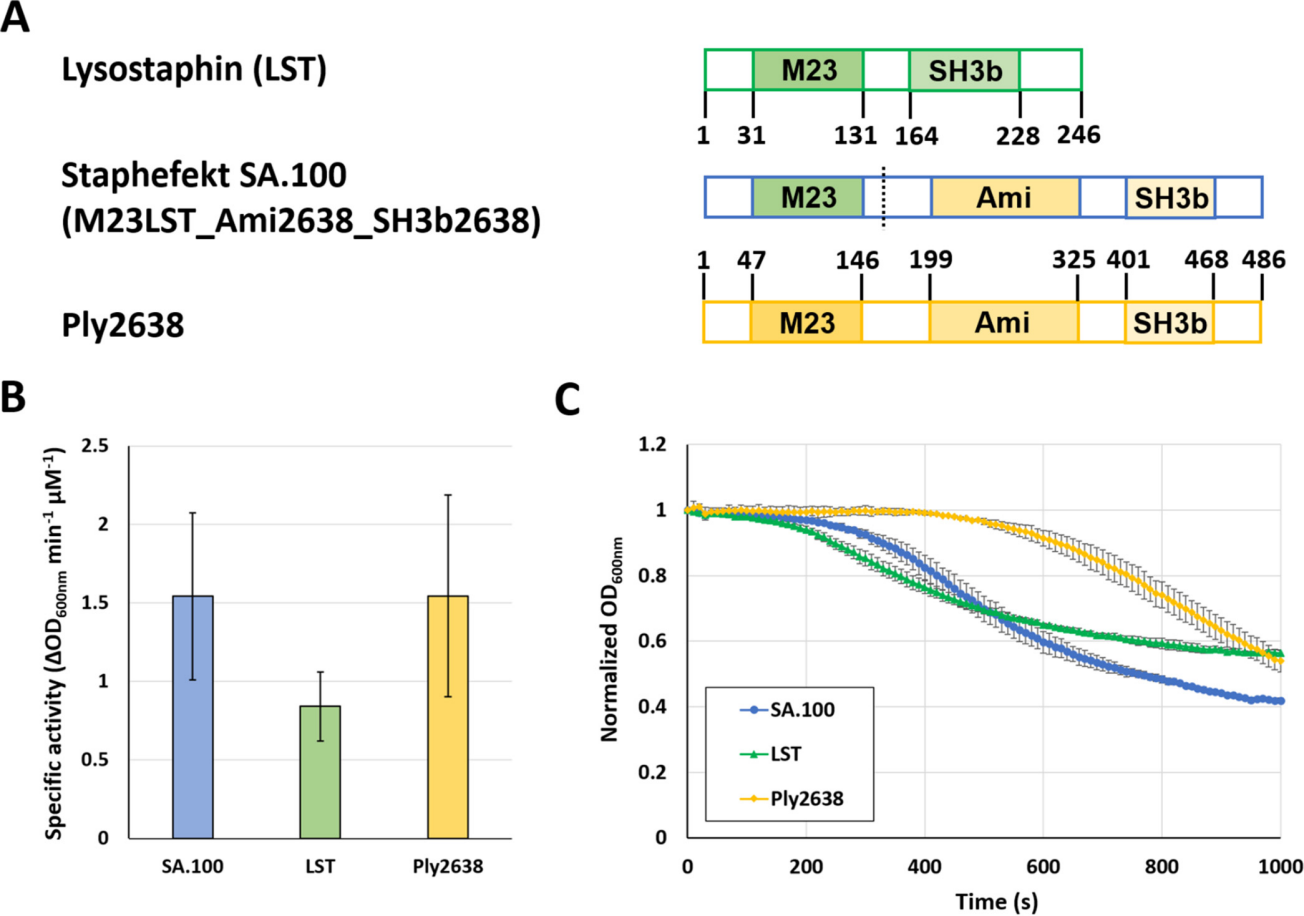
Modular architectures and lytic activities of staphylococcal phage endolysin Ply2638, lysostaphin, and the chimeric lysin Staphefekt SA.100 constructed from both enzymes. (A) Schematic representation of native and chimeric enzymes. Functional domains are represented by colored bars, and respective amino acid positions are indicated. The dashed line indicates the fusion site in the chimeric enzyme. M23, M23 endopeptidase domain; Ami, amidase domain; SH3b, SH3b cell wall binding domain. (B) Specific activities of parental and chimeric enzymes against S. aureus ATCC 12600, as determined by turbidity reduction assays. Specific activity values were determined from the steepest slopes of individual lysis curves (decrease in optical density over time) over a range of enzyme concentrations (12.5 to 100 nM). (C) Normalized and control-corrected lysis curves obtained with an enzyme concentration of 100 nM. Error bars represent SEM from 2 biological replicates, including 2 technical replicates each.

We created a chimeric lysin by substituting the M23 d-alanyl-glycine endopeptidase domain of the staphylococcal phage endolysin Ply2638 with the M23 glycyl-glycine endopeptidase domain of the potent Staphylococcus aureus-specific bacteriocin lysostaphin (LST). This resulted in the construct M23LST_Ami2638_SH3b2638, here named Staphefekt SA.100 (SA.100) (Fig. 1A). When we compared the parental and chimeric enzymes in turbidity reduction assays (TRAs) against live S. aureus cells in suspension, we found that SA.100 exhibits a similar specific activity to Ply2638 and is slightly more active than LST, even though this difference was not statistically significant (*P* > 0.05; ANOVA with *post hoc* Tukey honestly significant difference [HSD] test) (Fig. 1B). The specific activity is derived from the steepest slopes (change in optical density at 600 nm [ΔOD_600_]/min) of individual lysis curves (as exemplified in Fig. 1C), measured over a range of enzyme concentrations (12.5 to 100 nM, corresponding to 0.7 to 5.6 μg/mL in the case of SA.100). Interestingly, SA.100 displayed faster lysis kinetics than Ply2638, i.e., it caused an earlier onset of bacterial lysis, even though the steepest slopes of the two lysis curves were similar (Fig. 1C).

### A linker-improved version of SA.100 features improved antibacterial activity.

**FIG 2:**
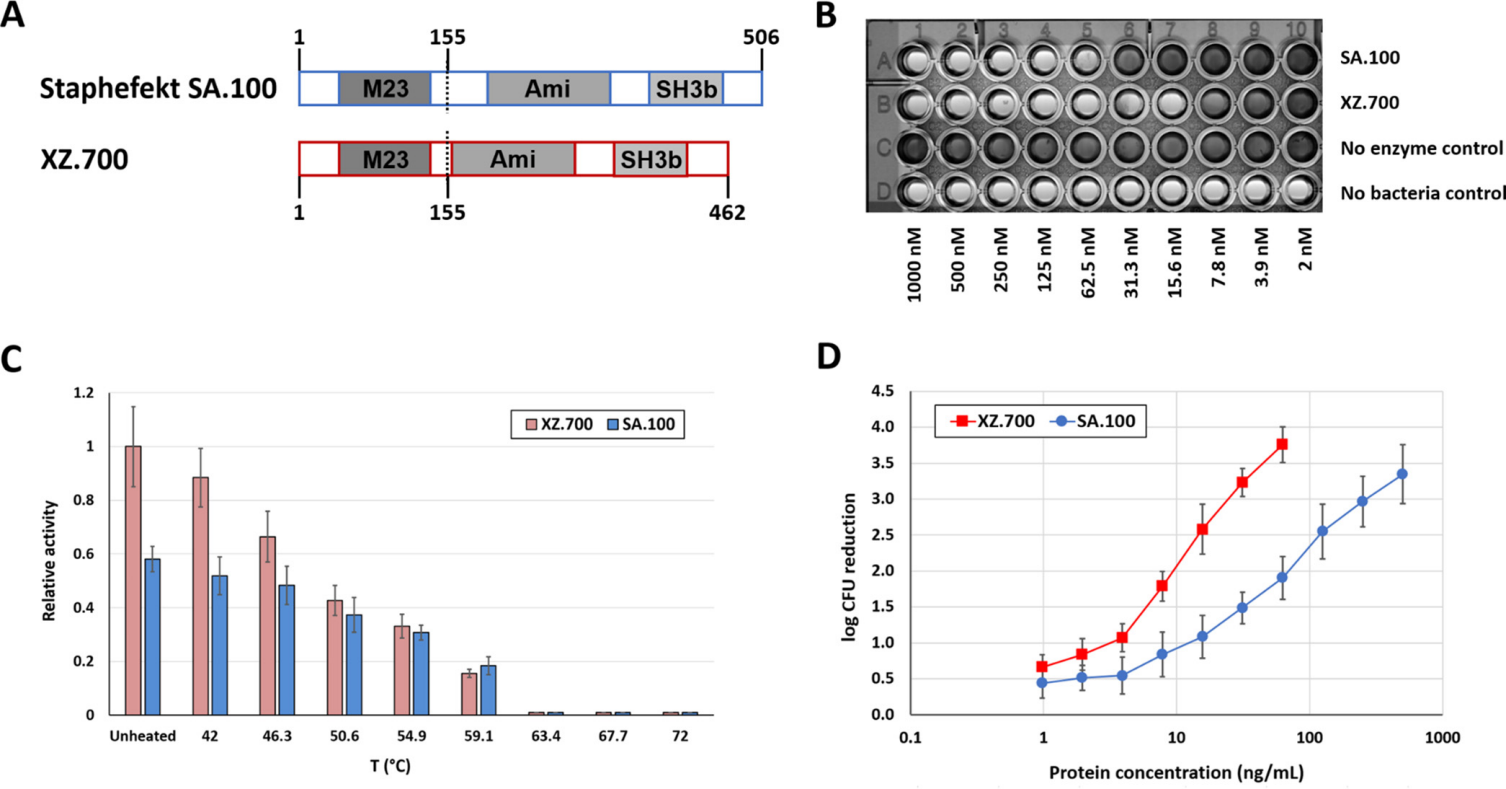
Comparison of anti-S. aureus activity of SA.100 and XZ.700. (A) Schematic representations of both constructs. Compared to SA.100, the 44-amino-acid linker region at the N terminus of the amidase domain of Ply2638 is absent in XZ.700. (B) Representative image of an MIC assay with both enzymes. MIC values were obtained by exposure of 2 × 10^5^ CFU to 3 × 10^5^ CFU of S. aureus 305 Newbould to serial dilutions of SA.100, XZ.700, or controls with and without bacteria. The image was taken after 20 h of incubation. The MIC values of Staphefekt SA.100 and XZ.700 were 62.5 nM (3.6 μg/mL) and 15.6 nM (0.8 μg/mL), respectively, in three independent assays. (C) Thermal stability of SA.100 and XZ.700. Proteins at 200 nM concentration were subjected to standard turbidity reduction assays against S. aureus SA113 after heat exposure for 10 min in PBS with subsequent cooling on ice. Error bars represent standard deviations from 3 biological replicates. (D) Quantitative killing assays with both enzymes. Log reductions of S. aureus ATCC 12600 cultures after exposure to the enzymes for 120 min in PBS-T are shown. The upper limit of detection was a 4-log reduction. Error bars represent standard deviations from at least 4 biological replicates.

Optimization of linker regions between individual functional domains of PGHs can substantially improve their activity (30). SA.100 contains an interdomain region consisting of two natural linkers at the C terminus of the LST-derived M23 domain and the N terminus of the Ply2638-derived amidase domain. In an attempt to further enhance the staphylolytic activity of SA.100 and based on learnings from our extensive engineering activities on staphylococcal PGHs in recent years (31–33), we created a linker-modified derivative of SA.100, which was named XZ.700 and features a deletion of a 44-amino-acid region at the N-terminal end of the Ply2638-derived protein fragment while retaining the same domain architecture (Fig. 2A; Fig. S1 in the supplemental material).

Direct comparison of both proteins in three different *in vitro* activity assays revealed that XZ.700 consistently displayed higher activity against S. aureus than SA.100 (Fig. 2B to D). When tested in MIC assays against a selection of MRSA and methicillin-susceptible S. aureus (MSSA) strains, the average obtained MIC values for SA.100 and XZ.700 were 19.96 μg/mL (∼350 nM) and 3.87 μg/mL (∼75 nM), respectively, with no obvious differences in susceptibility between MRSA and MSSA (Table 1; Fig. 2B). Of note, the only S. epidermidis strain tested within this experiment, ATCC 12228, proved insensitive to both enzymes (MIC > 128 μg/mL) (Table 1).

**TABLE 1:** MICs of SA.100 and XZ.700 against various staphylococcal strains^*a*^

Bacterial strain	MIC (μg/mL) of:	
	XZ.700	SA.100
S. aureus strains (MRSA)	2	16
** **130710015047		
** **130215015001	2	16
** **130403015176	16	64
** **130603015366	2	16
S. aureus strains (MSSA)		
** **305 (Newbould)	0.8	3.6
** **ATCC 29213	4	8
** **150825048101	4	16
** **150911021401	2	32
** **150918032701	2	8
** **305 (Newbould)		
S. epidermidis strain		
** **ATCC 12228	>128	>128

a *n* = 3 for strain 305 Newbould; *n* = 1 for all other strains.

Kinetic measurements in TRAs under standard conditions (i.e., in phosphate-buffered saline with 0.01% Tween 20 [PBS-T] at room temperature) revealed an approximately 70% increase in lytic activity of XZ.700 compared to SA.100. Thermodynamic stabilities as measured in the same assay format were found to be similar for both enzymes. When heated to increasing temperatures for 10 min prior to conducting TRAs under standard conditions, lytic activities of SA.100 and XZ.700 were gradually reduced, and they were completely abolished when temperatures exceeded 63°C (Fig. 2C).

The difference in activity between SA.100 and XZ.700 was most pronounced when they were compared in quantitative killing assays for their efficacy at reducing concentrations of viable S. aureus in suspension. Both enzymes reduced S. aureus numbers (CFU/mL) at concentrations as low as 1 ng/mL, and killing efficacies increased with increasing enzyme concentrations, with a higher rate observed for XZ.700 (Fig. 2D). At 15.6 ng/mL, XZ.700 caused a reduction in CFU/mL by >2.5 log units within 120 min, whereas an 8-fold higher concentration of SA.100 was needed to achieve a similar effect. These results corroborate the previous findings demonstrating a substantial improvement in staphylolytic activity of XZ.700 compared to SA.100 and led us to characterize XZ.700 in more detail.

### XZ.700 is specific for Staphylococcus aureus.

**FIG 3:**
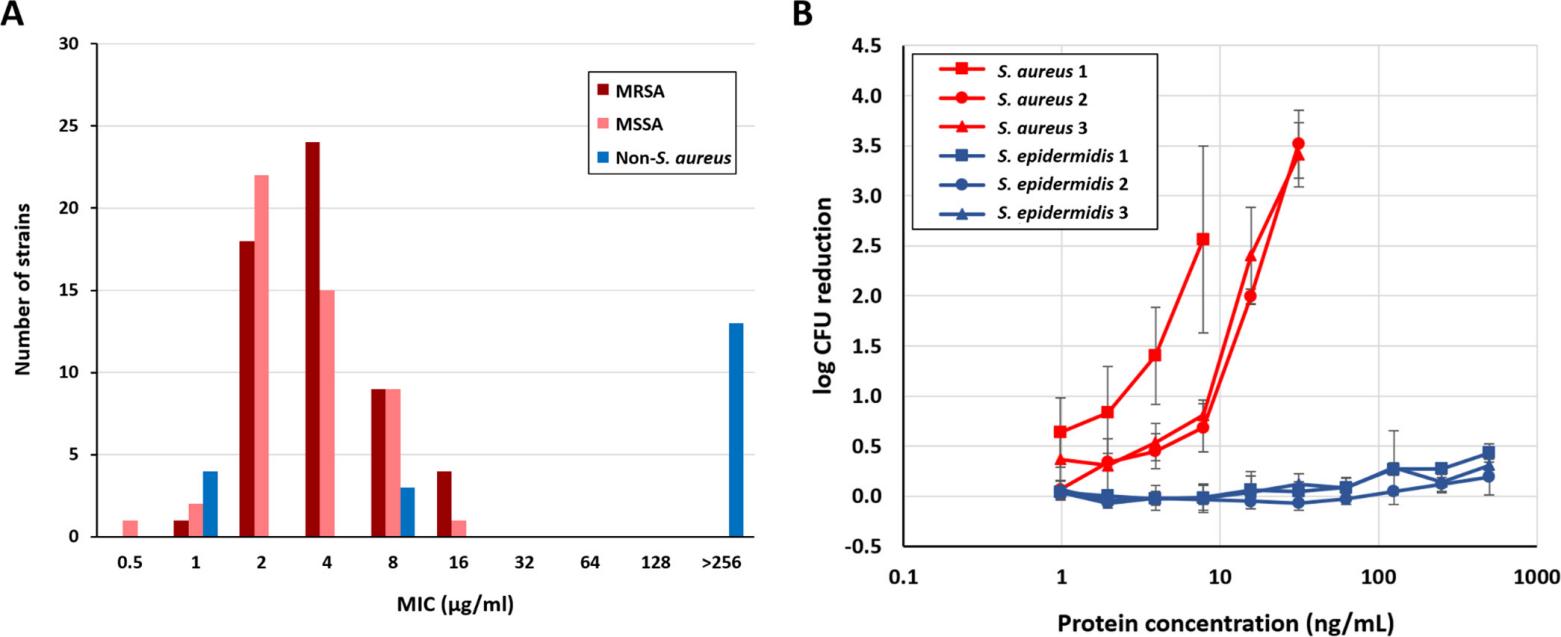
Specificity of XZ.700 for S. aureus. (A) MIC distribution of XZ.700 across 120 bacterial strains, including S. aureus (MSSA and MRSA) and non-S. aureus strains. (B) Quantitative killing assays with XZ.700 and multiple S. aureus (red) and S. epidermidis (blue) strains. S. aureus 1, strain 140122015197; S. aureus 2, strain 200309031601; S. aureus 3, strain 200224033101; S. epidermidis 1, strain 140712015198; S. epidermidis 2, strain 200414016601; S. epidermidis 3, strain 200331020501. Error bars represent standard deviations from 3 biological replicates.

To provide more evidence for the specificity of XZ.700 for S. aureus, MICs were determined against a comprehensive collection of bacterial strains from different geographic regions, including 50 MRSA strains, 50 MSSA strains, and 20 non-S. aureus strains. The latter consisted of 17 staphylococcal species other than S. aureus and 3 nonstaphylococcal organisms (Corynebacterium jeikeium, Cutibacterium acnes, and Saccharomyces cerevisiae) (Table S1). For the tested S. aureus isolates (MSSA and MRSA), MICs ranged from 0.5 to 16 μg/mL of XZ.700 (Fig. 3A). The distribution of MICs did not significantly differ between MSSA and MRSA strains (median MICs of 2 and 4 μg/mL, respectively; *P* = 0.11, independent-samples *t* test), and also the mean MIC values of the MSSA and MRSA populations (3.89 ± 2.88 and 4.90 ± 3.88, respectively) were not significantly different from each other (*P* = 0.14). None of the tested S. aureus strains showed intrinsic resistance to XZ.700. Importantly, of the 17 staphylococcal non-S. aureus strains, 10 strains did not show any susceptibility to XZ.700 activity (MICs > 256 μg/mL; Table S1). Most of the strains that were susceptible to the enzyme represent species closely related to S. aureus, sharing similar virulence factors and/or causing similar infections in humans or other mammalian species (34–36). These included three Staphylococcus pseudintermedius strains isolated from wound infections in dogs and one Staphylococcus chromogenes strain isolated from bovine mastitis, all showing an MIC of <2 μg/mL. Furthermore, three Staphylococcus lugdunensis strains isolated from clinical samples of different human infections showed an MIC of 8 μg/mL. The other coagulase-negative staphylococcal species, including S. epidermidis and the nonstaphylococcal control strains, all were not susceptible to XZ.700-mediated killing (Table S1). The specificity of XZ.700 for S. aureus was further demonstrated in quantitative killing assays, using three S. aureus and three S. epidermidis strains (Fig. 3B). While XZ.700 showed concentration-dependent killing of all tested S. aureus strains, no measurable activity was detected against the three S. epidermidis strains.

**FIG 4:**
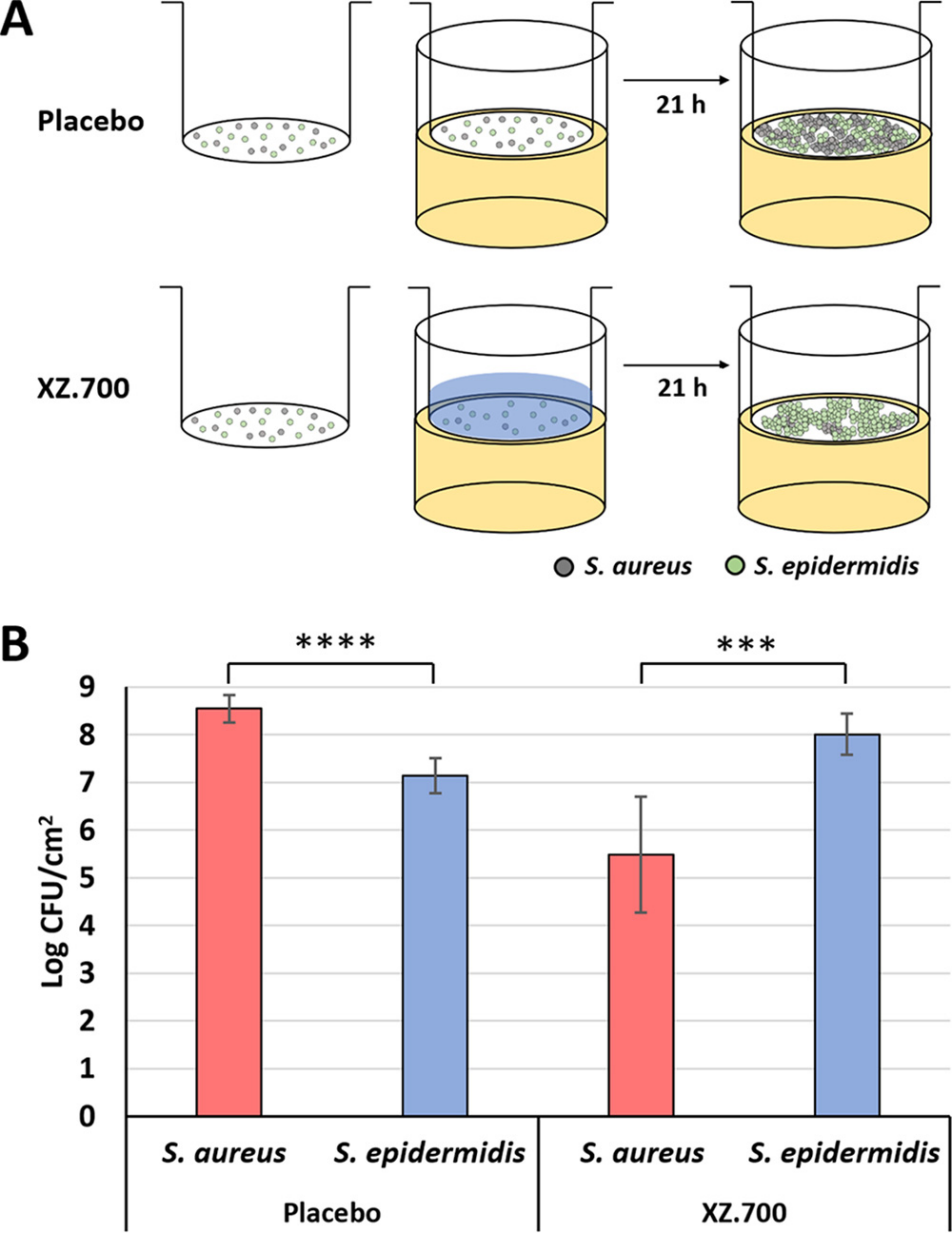
Selective killing of S. aureus by XZ.700 in mixed surface-adhered staphylococcal populations. (A) A mixed culture of S. aureus and S. epidermidis was grown on transwell membranes (∼10^4^ CFU/cm^2^ of a 1:1 mixture at the time of seeding) inserted into agar-filled wells for 24 h in total. Two hours after seeding, the adhered bacteria were exposed to XZ.700 solution or a control treatment (buffer) for 1 h before being further incubated for 21 h in the absence of the enzyme solution/buffer. (B) Concentrations of S. aureus and S. epidermidis on transwell membranes 21 h after treatment with buffer (placebo) or 100 μg/mL of XZ.700. Error bars represent standard deviations from 6 independent experiments. ****, *P* < 0.0001; ***, *P* < 0.001.

To further substantiate the specificity of XZ.700 for S. aureus, we grew mixed cultures of S. aureus and S. epidermidis on transwell membranes (inoculated at a ratio of 1:1), mimicking a simplified skin microbiome (Fig. 4A). After 2 h of growth, the surface-adhered cultures were treated with 100 μg/mL of XZ.700 in PBS-T or PBS-T alone as a placebo control for 1 h, after which the liquid was removed. Following further incubation for 21 h in the absence of any treatment, bacterial cells were harvested and S. aureus and S. epidermidis numbers determined. As shown in Fig. 4B, S. aureus overgrew S. epidermidis by approximately 1.5 log units (ratio, 25:1) in the placebo control within 24 h of incubation (*P* < 0.0001; unpaired *t* test). However, in the XZ.700-treated samples, S. aureus concentrations were found to be 2.5 log units below those of S. epidermidis (ratio, 1:335) at the end of the experiment (*P* < 0.001). This suggests that XZ.700 selectively kills S. aureus in mixed surface-adhered bacterial cultures while allowing S. epidermidis to grow.

### XZ.700 does not induce resistance in S. aureus.

**FIG 5:**
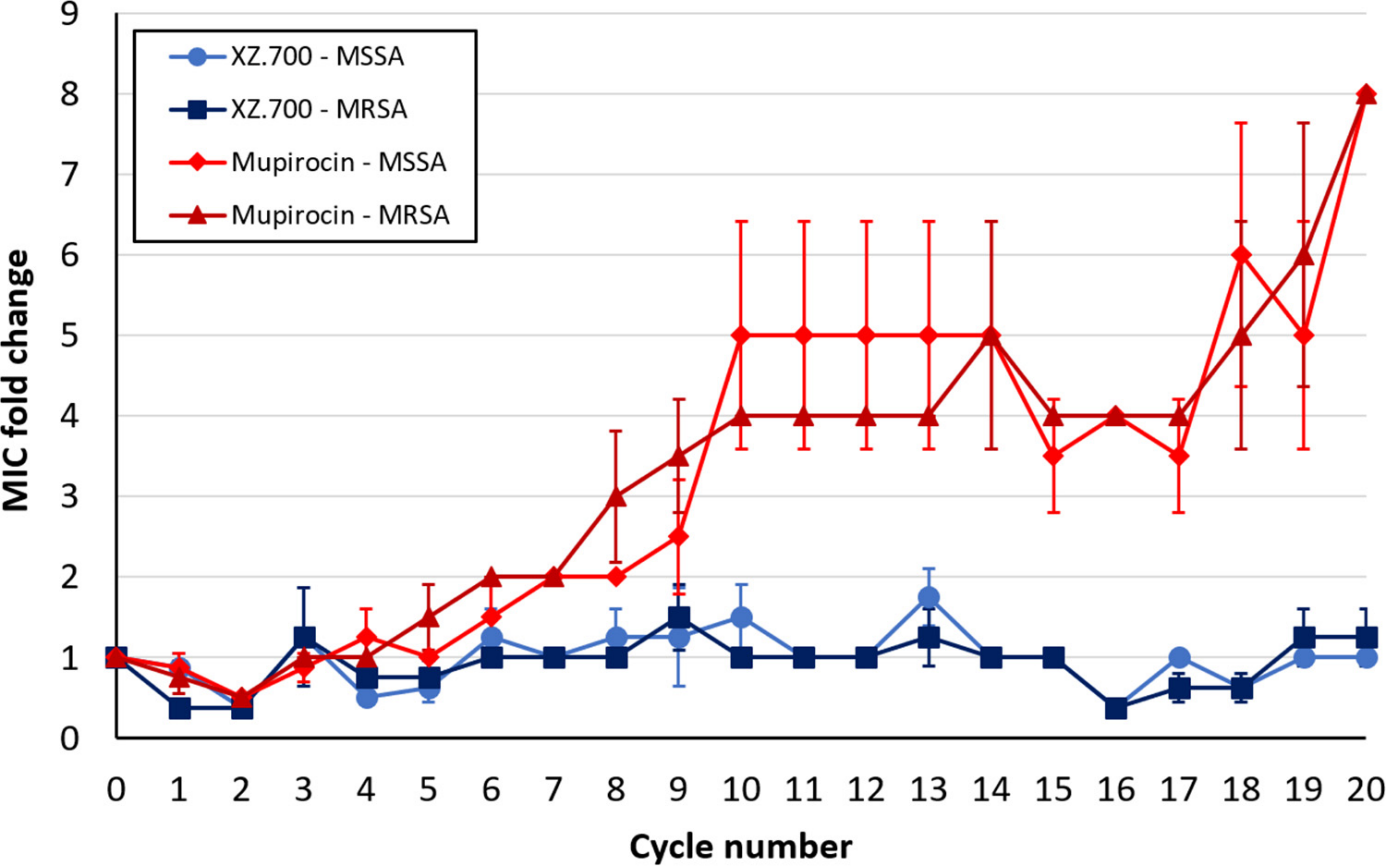
Fold changes in XZ.700 and mupirocin MICs against MSSA and MRSA strains over 20 cycles of repeated exposure to sub-MICs. Each curve represents mean values and SEMs from 2 strains tested in duplicate. Strains used for this experiment included ATCC 29213 (MSSA) and the clinical isolates 170606037501 (MSSA), 130603015366 (MRSA), and 150212029501 (MRSA).

Unlike classical antibiotics or the bacteriocin LST, endolysins have been suggested to be refractory to resistance development in bacteria due to their highly conserved binding and cleavage sites in the peptidoglycan (16). XZ.700 is derived from the phage endolysin Ply2638 but also contains one catalytic domain from LST. This being said, we set out to investigate whether XZ.700 could provoke resistance in S. aureus after several rounds of repeated exposure to sub-MICs. Mupirocin, an antibiotic widely used as a topical agent against superficial S. aureus infections or colonization of skin and mucous membranes, was included in these experiments for comparison. After 20 cycles of repeated exposure, no induction of resistance was observed in either MSSA or MRSA strains against XZ.700, while MICs of mupirocin gradually increased to 8 times the original MIC in all strains (Fig. 5). Although the MICs for XZ.700 fluctuated by one 2-fold dilution step compared to the starting MIC, no gradual increase of MICs was observed. At cycle 20, the difference in MIC fold changes between XZ.700 and mupirocin across all tested strains was statistically significant (*P* < 0.0001; unpaired *t* test).

### XZ.700 in solution or formulated as a cream reduces numbers of viable MRSA on reconstituted human epidermis.

**FIG 6:**
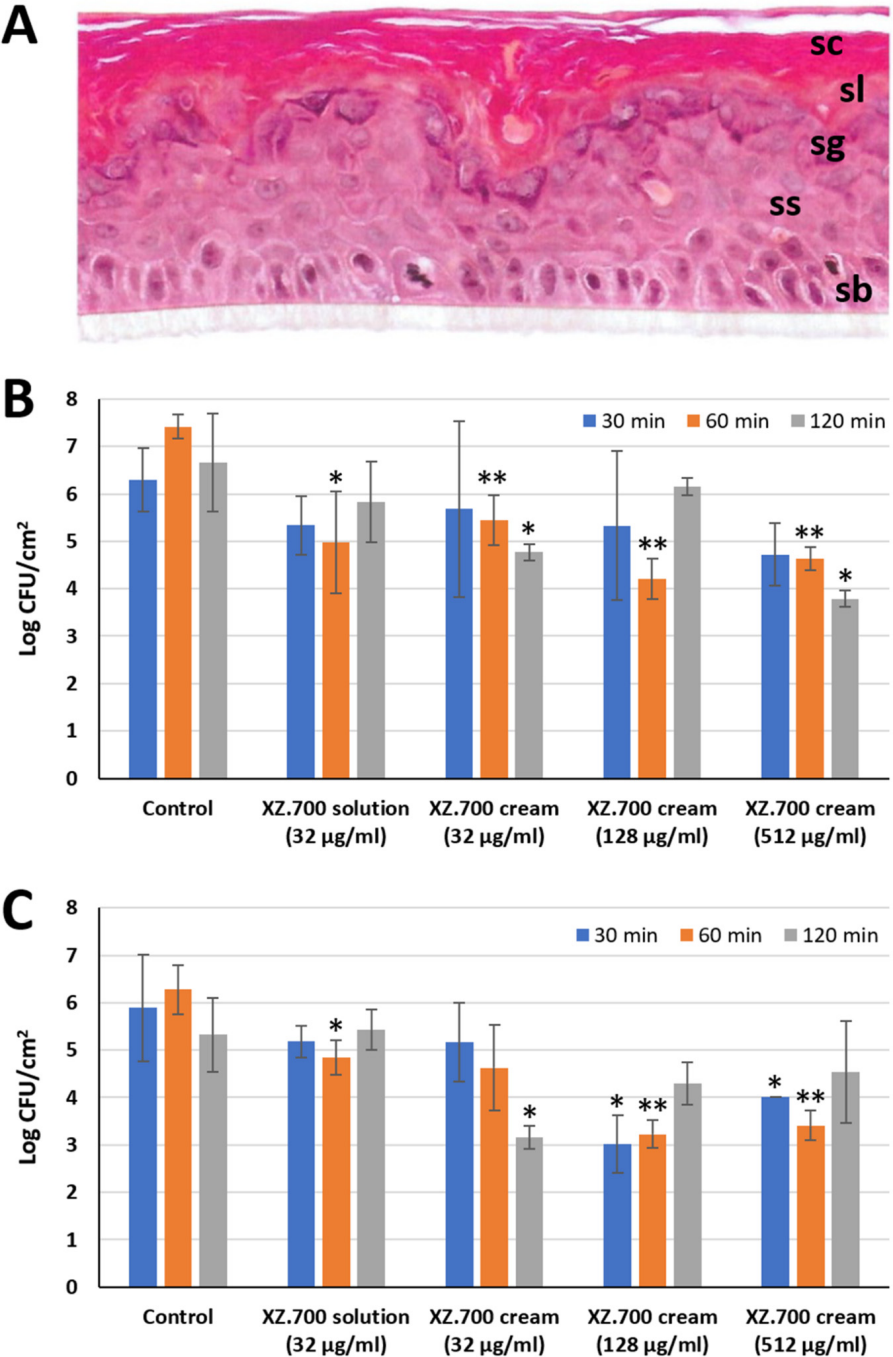
Activity of XZ.700 in solution and cream formulation against MRSA on reconstituted human epidermis (RHE). (A) HES-stained vertical paraffin section of RHE used in these experiments, with stratum corneum (sc), stratum lucidum (sl), stratum granulosum (sg), stratum spinosum (ss), and stratum basale (sb) visible. (B and C) Concentrations of viable nonadherent (B) and adherent (C) S. aureus ATCC 33591 (MRSA) after treatment of colonized RHE with XZ.700 in solution or cream formulation at different concentrations for 30, 60, or 120 min. Bacterial concentrations after treatment significantly different from the respective untreated controls are marked with asterisks (*, *P* < 0.05; **, *P* < 0.01). Error bars represent standard deviations from 3 biological replicates. The used enzyme concentrations of 32, 128, and 512 μg/mL correspond to 0.96, 3.84, and 15.36 μg/cm^2^ RHE, respectively.

The absence of resistance development and the specificity of XZ.700 for S. aureus make this enzyme a promising novel antibacterial candidate agent for the treatment of S. aureus infections in which the preservation of a healthy microbiome is desired, such as in skin infections. To evaluate the potential of XZ.700 as an agent to decrease the S. aureus burden on colonized or infected human skin, we tested the enzyme in solution and formulated as a cream on MRSA-colonized reconstituted human epidermis (RHE). The RHE samples we obtained for this purpose had been cultivated from human keratinocytes for 17 days and were fully differentiated, with stratum corneum, stratum lucidum, stratum granulosum, stratum spinosum, and stratum basale visible in hematoxylin-eosin saffron (HES)-stained cross-sections (Fig. 6A). On the day of the experiment, the RHE samples immersed in antibiotic-free maintenance medium were inoculated with 6 × 10^6^ CFU/mL of S. aureus ATCC 33591 (MRSA; MIC of XZ.700, 0.50 ± 0.27 μg/mL). Following incubation at 35°C for 4 h, the medium was removed and the colonized RHE treated with XZ.700 in solution (32 μg/mL; 64-fold MIC) or a cream formulation at three different concentrations (32 μg/mL, 128 μg/mL, 512 μg/mL) for 30, 60, or 120 min or left untreated as a control. Residual viable S. aureus cells after the treatment were determined both in the apical fraction (nonadhered bacteria) and the homogenized tissue sample (adhered bacteria). As shown in Fig. 6B, the 60-min treatments were most effective at reducing nonadherent S. aureus numbers for both XZ.700 solution and the different cream formulations. While there was a trend toward higher efficacy with increasing concentrations in the cream, these differences were not statistically significant. The most effective treatment was the XZ.700 cream at 128 μg/mL applied for 60 min, resulting in a significant 2-log reduction compared to the untreated control (*P* < 0.01). In contrast, none of the 30-min treatments led to a significant reduction in bacterial numbers. Similar observations were made for the adherent cells in the tissue homogenates (Fig. 6C). Here, the concentration-dependent effect was more pronounced, with XZ.700 cream at 128 μg/mL and 512 μg/mL, applied for 60 min, causing the highest reduction in adhered S. aureus numbers compared to the control (approximately 3 log units; *P* < 0.01). Overall, these results demonstrate that XZ.700 is effective against S. aureus also in more complex environments such as reconstituted tissues and in cream formulations, as they are commonly used for topical applications on skin.

### XZ.700 gel reduces S. aureus numbers in a murine skin infection model.

To evaluate the anti-S. aureus activity of XZ.700 also *in vivo*, we employed a mouse model of superficial skin infection combined with an *in vivo* bioluminescence imaging system. In a preliminary experiment, we monitored the concentration of a bioluminescent S. aureus USA300 strain (MRSA) on tape-stripped skin of female and male mice over a period of 79 h following initial inoculation with 10^8^ CFU/animal. For this purpose, bioluminescence produced by viable bacteria was recorded at multiple time points during the entire 3-day experiment, and the resulting color-scale images were overlaid with grayscale photographs of the immobilized mice (Fig. S2A). Furthermore, bioluminescence was quantified and plotted over time as relative intensity (Fig. S2B). We found that the intensity of the bioluminescence signal (corresponding to the bacterial load on the murine skin; Fig. S3) decreased by around 42% in males within the first 8 h after infection, whereas in females, it remained unchanged or even slightly increased during the same period. Overall, male mice showed a markedly faster clearance of bacteria than female mice, with percent intensity values significantly different between the two groups at time (*t*) of 8 h (*P* = 0.02). This was also reflected in the number of viable S. aureus cells obtained from homogenized skin biopsy specimens at the end of the experiment. While the bacterial concentration on male skin was 1.6 ± 0.7 × 10^5^ CFU/cm^2^, it was more than 2-fold higher in female mice (3.6 ± 1.5 × 10^5^ CFU/cm^2^), although this difference was not statistically significant (Fig. S2C). Overall, the results obtained from this preliminary study suggest that female mice are less efficient at clearing S. aureus infection on tape-stripped skin than male mice, for which reason we chose female mice for the following infection and treatment study.

**FIG 7:**
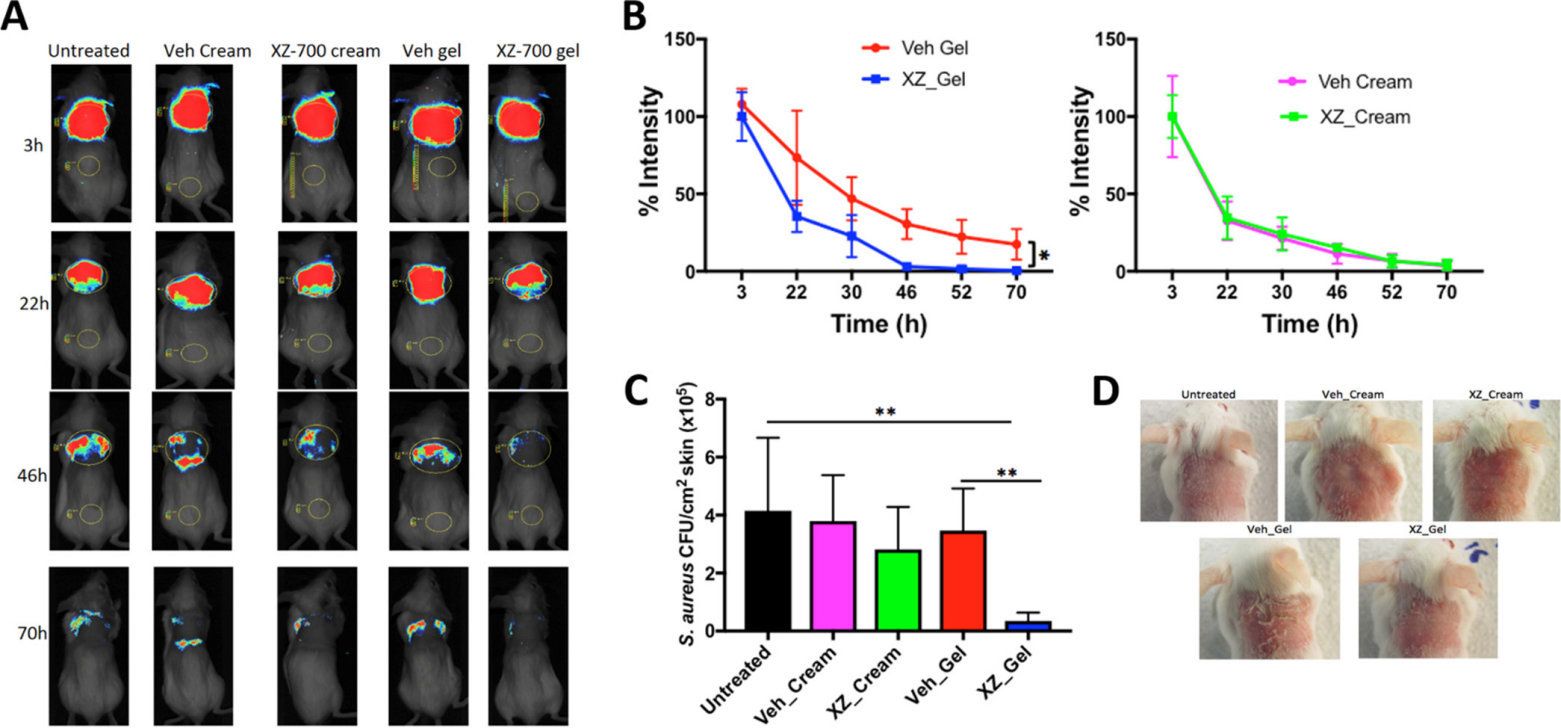
Efficacy of XZ.700 formulated as cream or gel in a mouse model of S. aureus skin infection. (A) Representative bioluminescence images of tape-stripped mice infected with the bioluminescent S. aureus strain USA300 LAC::*lux* and treated with XZ.700 formulated as cream or gel (30 μg XZ.700 per mL cream or gel) or the respective vehicle controls (Veh). (B) Luminescence intensity measured over time in infected animals treated with XZ.700 (XZ) gel (left) or cream (right) or the respective vehicle controls (Veh) (*n* = 6). (C) S. aureus CFU counts/cm^2^ on murine skin left untreated or undergoing various treatments as described in panels A and B 74 h after infection (*n* = 6). (D) Representative images showing the appearance of murine skin 74 h after infection with S. aureus and various treatments as described above.

Similar to what has been described above, mice were infected with 10^8^ CFU of the bioluminescent S. aureus on tape-stripped skin, and the infected areas (around 2 cm^2^) were treated with XZ.700 formulated as gel or cream or corresponding vehicle controls (gel or cream without enzyme) 6 times during a period of 70 h, with the first treatment applied at 3 h postinfection. The amounts of cream and gel applied per animal during the entire experiment were approximately 30 mg and 25 mg, respectively. Bioluminescence imaging revealed that topical treatment with XZ.700 gel significantly enhanced the clearance of S. aureus on tape-stripped skin compared with untreated mice or vehicle control animals, whereas treatment with XZ.700 cream resulted in a similar clearance as observed in untreated or vehicle cream-treated mice (Fig. 7A and B). The luminescence intensity on XZ.700 gel-treated animals was significantly lower than on the respective vehicle control mice at the end of the experiment (*P* < 0.05; Fig. 7B). These results were confirmed by enumeration of S. aureus on skin homogenates 74 h after the infection (Fig. 7C). While the reduction in CFU/cm^2^ skin in XZ.700 cream-treated animals compared to untreated or cream vehicle controls was not statistically significant, XZ.700 gel caused a significant reduction compared to the respective untreated and gel vehicle controls (*P* < 0.01). We observed very weak redness in almost all mice before the cutaneous S. aureus infection, consistent with the mechanical injury induced by tape stripping. This redness was not worsened by the infection and disappeared 48 h after infection. For some mice treated with vehicle gel, we observed slight skin dryness starting at 36 h and remaining until 72 h after the infection, whereas this was not observed in mice treated with XZ.700 gel (Fig. 7D).

## DISCUSSION

Novel classes of antibacterial agents that are specific for the target pathogen and refractory to resistance development are highly desirable, particularly in the light of the increasing prevalence of antibiotic-resistant strains worldwide, which represents one of the most urgent problems of our time (37). One of the biggest disadvantages of using conventional antibiotics for the treatment of bacterial infections, besides their high chance of inducing resistance, is their broad-range detrimental effect on commensal bacterial populations. This particularly applies to the treatment of skin infections which are caused or aggravated by S. aureus, such as AD, where any off-target activity against beneficial microorganisms of the healthy skin microflora should be avoided. The two chimeric PGHs described here and, in particular, XZ.700 due to its enhanced lytic activity against S. aureus compared to SA.100, are promising candidates for such applications. SA.100, the modular design and construction of which is described here for the first time, has previously been used successfully in a cetomacrogol-based cream formulation for the treatment of chronic and recurrent S. aureus-related dermatoses in three single-patient case studies (38). All three patients were suffering from skin conditions (folliculitis or impetigo) associated with S. aureus infection and had been treated unsuccessfully with antibiotics prior to the start of SA.100 treatment. Importantly, no induction of resistance to SA.100, as determined by MIC assays with S. aureus isolates obtained from one of the patients, was observed despite repeated application of the endolysin (up to 2 times daily for several months) (38). In another study, SA.100 in the same cetomacrogol cream induced a clinically relevant and statistically significant improvement of both severity scores, as well as quality of life, in 43 patients with atopic dermatitis (39).

Besides antibiotics, also, LST is known to be prone to resistance development. This is because this bacteriocin cleaves within the pentaglycine bridge, which is the most variable part of S. aureus PG, whereas all staphylococcal phage endolysins described to date target more conserved portions of the PG (20). Various S. aureus mutant strains featuring alterations within the pentaglycine bridge and, consequently, reduced susceptibility to LST have been described (40). This being said, our finding that XZ.700 does not induce resistance in MRSA and MSSA strains during repeated cycles of exposure is highly encouraging and in agreement with previous findings on SA.100 (38). In contrast, increases in MIC of more than 500-fold had been observed in similar experiments with LST (41). The absence of resistance induction in SA.100 and XZ.700 can likely be attributed to the combination of two different EADs (i.e., a Gly-Gly endopeptidase and a MurNAc-l-Ala amidase) within the same molecule. In this case, two simultaneous independent mutations within the PG would be required to render the strains resistant, which is highly unlikely to occur. This is also in agreement with previous research showing that the rate of resistance development against staphylococcal PGHs decreases with an increasing number of different EADs within the same enzyme (41). Similar to XZ.700, the chimeric PGH constructs K-L and L-K described in this study contain an LST-derived M23 domain besides two other EADs originating from a phage endolysin. Despite the demonstrated functionality of the M23 domain in the context of these chimeras (via mass spectrometry analysis of digested PG), K-L and L-K displayed 73- to 291-fold reduced fold changes in MICs compared to LST when tested in repeated-exposure experiments. This being said, it would be interesting to investigate whether both EADs included in XZ.700 retain their catalytic activity, e.g., via PG digestion product analysis or active-site knockouts. Further research could also include testing bacteria repeatedly exposed to XZ.700 for their susceptibility to LST and determining the activity of XZ.700 against known LST-resistant strains.

The replacement of the native Ply2638 M23 endopeptidase domain by the (pentaglycine bridge-specific) M23 domain of LST in SA.100 and XZ.700 was expected to shift the specificity of these enzymes toward S. aureus. However, the high degree of specificity observed for XZ.700 (illustrated by MICs >256 μg/mL and absence of any measurable activity against S. epidermidis in quantitative killing assays) (Fig. 3; Table S1) was rather surprising given the highly conserved PG cleavage site of its Ply2638-derived amidase domain (MurNAc-l-Ala) (20), which is also present in S. epidermidis (28). This may argue for a substantially higher contribution of the M23 domain to the overall activity of XZ.700 than the amidase domain. Alternatively, this effect could be explained by the possibility that, despite its highly conserved cleavage site, also, the amidase domain exhibits a certain preference for S. aureus PG by recognizing and binding to a larger portion of the PG structure, extending beyond the actual scissile bond and including parts of the pentaglycine bridge. This specificity could be further enhanced by the presence of the SH3b binding domain in direct proximity of the amidase domain. The SH3b cell wall-targeting domain of LST has been demonstrated to require an intact pentaglycine bridge (as only present in S. aureus) to exhibit its full binding capacity (42–44), and, given the high degree of conservation of SH3b domains throughout all known staphylococcal PGHs (45), the same has been suggested for SH3b domains derived from staphylococcal phage endolysins (46). The observation that the S. aureus specificity of XZ.700 does not only hold true in experiments with individual bacterial strains but also in our mock microbiome study in the presence of S. epidermidis (Fig. 4) is highly encouraging in light of a potential application of this enzyme on human skin as a microbiome-friendly anti-S. aureus agent. There is a broad consensus that S. aureus plays an important role in aggravating disease in AD patients (10, 14, 47). While, also, S. epidermidis can cause infections in humans (48), there is currently more evidence for its protective effect within the skin microbiome (e.g., by protecting against infection by pathogenic bacteria, promoting wound repair, tuning skin immunity, and protecting against skin tumors) than for its role as an aggravator in AD (49, 50). Importantly, we observed a shift in our mixed staphylococcal culture toward S. epidermidis 21 h after the end of the XZ.700 treatment, suggesting that the enzyme might be capable of exerting a long-term positive effect on a skin microbiome. However, it is important to acknowledge the limitations of this simplified microbiome model, which consists of only two bacterial species and therefore does not take into account possible effects of other skin microbiota on the microbiome during endolysin treatment. Therefore, these experiments should be followed up by studies employing more complex microbial compositions or even full skin microbiomes.

The comparative *in vitro* characterization of our two chimeric lysins revealed that XZ.700 outperforms SA.100 in three independent activity assays, i.e., MICs, TRAs, and quantitative killing assays (qKAs) (Fig. 2). High thermostability has previously been reported for individual phage-derived lytic enzymes, such as the virion-associated PGH gp36C of Pseudomonas aeruginosa phage phiKMV (51) or the *Listeria* phage endolysin PlyP35, which retained considerable lytic activity even after heating to 90°C (52). While SA.100 and XZ.700 exhibited lower thermostability, both enzymes retained approximately 90% of their activity after exposure to 42°C, which is higher than normal human skin temperature by 5 to 9°C, suggesting sufficient stability for skin applications. Also under these conditions, the activity of XZ.700 surpassed that of SA.100. Taken together, these results clearly demonstrate that XZ.700 is the more potent enzyme, even though results from the different assays may differ on a quantitative level. Similar quantitative differences have been reported for other lytic enzymes that have been compared in multiple activity assays (20, 41, 53), and this observation has been explained with differences in sensitivity between these assays and assay-specific inherent biases, each of them favoring enzymes with certain biochemical properties such as regarding molecule size, charge, or hydrophobicity (16). The increased activity of XZ.700 compared to SA.100 can most likely be attributed to the deletion of a Ply2638-derived linker region in XZ.700, which represents the only difference between the two enzymes. The potential impact of interdomain linkers on lytic activity of (chimeric) endolysins has been recognized in earlier studies (30, 54, 55), with the exchange or variation of such linkers being able to either boost, decrease, or even completely abolish the activity of the respective enzymes (30). The likely reason for this is that the length, structure, and flexibility of the linker peptide determines the geometric orientation of the two adjacent functional domains relative to each other and, therefore, their degree of alignment with the respective cleavage or binding sites in the PG (30, 54).

Besides its high activity *in vitro* against planktonic and adhered S. aureus cells, as demonstrated in this study, XZ.700 has recently been reported to also effectively degrade MRSA biofilms grown on titanium surfaces under static and dynamic conditions for 24 or 48 h (56). This antibiofilm activity, which has also been described for several other PGHs (19, 57, 58), is an important feature when considering the application of such lytic enzymes as antibacterial agents, given the prominent role of biofilms in bacterial infections and their resilience to conventional treatment regimens (6, 22, 59). One reason for this observed resilience is that bacteria within such biofilm communities frequently exist as persisters, i.e., dormant, nondividing cells, which display high tolerance to many antibacterial agents (60). By attacking the bacterial PG, PGHs exhibit a mode of action that is independent of the metabolic state of the cell, which is in agreement with previous reports demonstrating activity of PGHs against persister cells (19, 61). Besides biofilms, dormant bacteria also exist in abscesses and intracellular compartments (5). A recent study reported activity of PGHs fused to cell-penetrating peptides against S. aureus residing intracellularly within various cell lines and within abscesses in a mouse model (31).

The promising *in vitro* results with XZ.700 obtained here and in the aforementioned biofilm study are corroborated by our findings in the reconstituted human epidermis model and the mouse model of superficial skin infection. Reconstructed human epithelia models have been described to closely mimic *in vivo* human tissues in terms of morphological (i.e., the presence of a multistratified epithelium), biochemical, and physiological properties and therefore currently represent the most promising alternative to animal models, *ex vivo* explants, and submerged cell monolayers for safety and efficacy evaluation of topically applied agents (62, 63). Here, we demonstrated that XZ.700 can significantly reduce nonadherent and adherent S. aureus numbers in a complex environment mimicking human skin when formulated as a solution or a cetomacrogol-based cream (Fig. 6). Of note, a similar cream formulation with SA.100 as the main ingredient had previously proven safe in human clinical trials (11, 12). Interestingly, the 60-min treatments with XZ.700 led to significant reductions in bacterial numbers in 7 out of 8 cases, whereas 120 min treatments significantly reduced the numbers in only 3 out of 8 cases. One can speculate if this could possibly indicate a relatively fast inactivation of the enzyme under the applied experimental conditions or it could, rather, be attributed to the inherent variability of the experiment. In any case, this question warrants further investigation in view of the intended application of XZ.700 on human skin. Contrary to our observations in the reconstituted human epidermis model, application of an identical XZ.700 cream did not significantly reduce S. aureus numbers compared to the untreated and vehicle controls in our murine skin infection model (Fig. 7C). In this experimental setup, the gel formulation of XZ.700 proved significantly more effective at reducing bacterial numbers on the skin than the cream. This difference can likely be explained by cream- and gel-specific matrix effects, with the gel formulation allowing for a more efficient release of the enzyme from the matrix and, consequently, a higher local concentration of active enzyme on the treated skin. Interestingly, we found that, in the absence of XZ.700 treatment, male mice cleared S. aureus from their skin significantly faster than female mice. Gender bias in bacterial infections (including those caused by S. aureus) has been reported previously, with the direction of the bias depending on the pathogen and the site of infection (64, 65). It remains unclear if the gender-specific difference observed here can have an impact on XZ.700 efficacy. While the results of our murine skin infection model are encouraging by demonstrating efficacy of XZ.700 in a complex *in vivo* setting, one should keep in mind the limitations of murine models from a translational point of view. In fact, there are important differences in skin morphology (e.g., regarding skin thickness, the number of epidermal layers, and adhesion to underlying tissues) and wound-healing mechanisms between mice and humans (66). This being said, future research should focus on evaluating the efficacy of XZ.700 also in larger animal models such as pigs, which are more suitable to mimic the situation in human skin (67).

Overall, the results from our study corroborate the high potential of PGHs in general as a novel class of antibacterial agents and of XZ.700 in particular for the treatment of S. aureus-induced or -aggravated skin conditions due to its unique specificity for S. aureus, its high antibacterial activity, and the absence of resistance induction against this enzyme.

## MATERIALS AND METHODS

### Bacterial strains and culture conditions.

Staphylococcus aureus strains ATCC 12600 and SA113 (68) were used for turbidity reduction assays (TRAs) and quantitative killing assays (qKAs) conducted in this study. In addition, the following clinical isolates available in the strain collection of the Regional Public Health Laboratory Kennemerland were used for comparison of killing activity against S. aureus and S. epidermidis: S. aureus strains 140122015197 (blepharitis isolate), 200309031601 (skin isolate from acne patient), and 200224033101 (skin isolate from rosacea patient) and S. epidermidis strains 140712015198 (blepharitis isolate), 200414016601 (human blood isolate), and 200331020501 (human skin isolate). S. aureus ATTC 12600 and S. epidermidis strain 140712015198 were also used for mixed-culture transwell experiments. Strains used for MIC assays are listed in Table 1 and Table S1 in the supplemental material. ATCC 29213 (MSSA) and the clinical isolates 170606037501 (MSSA; isolate from prosthetic joint infection), 130603015366 (methicillin-resistant S. aureus [MRSA]; isolate from nasal colonization screening), and 150212029501 (MRSA; isolate from furunculosis) were used for resistance induction experiments. S. aureus strain ATCC 33591 was used for experiments with RHE and the bioluminescent MRSA strain USA300 LAC::*lux* (69) for the murine skin infection model.

Staphylococcal strains were grown in tryptic soy broth (TSB) or brain heart infusion (BHI) at 37°C or 35°C. Escherichia coli strains were cultured in Luria-Bertani (LB) medium supplemented with appropriate antibiotics for plasmid selection.

### DNA techniques and cloning procedures.

Standard molecular cloning techniques (70) were used to generate plasmid constructs encoding recombinant endolysins. In the first round of cloning, the endolysin gene ply2638 identified in Staphylococcus phage 2638a (71) was inserted into BamHI and SalI sites of pQE-30 (Qiagen), resulting in pHPL2638. Its heterologous expression in E. coli results in an N-terminally 6×His-tagged version of Ply2638 (HPL2638) that can be purified by immobilized metal ion affinity chromatography (IMAC). In a similar manner, a construct encoding an N-terminally His-tagged version of mature lysostaphin (lysostaphin fragment Ala248-Lys493) (72) was generated (pHLST). A truncated variant of Ply2638 lacking the N-terminal M23 domain, pHAmi2638_SH3b2638, was created by inserting a *ply*2638 fragment corresponding to Leu138-Lys486 into SacI-SalI sites of pQE-30. A construct encoding an N-terminally His-tagged version of a fusion protein subsequently named Staphefekt SA.100 (pHM23LST_Ami2638_SH3b2638; SA100) was then obtained by inserting a fragment corresponding to the M23 domain of lysostaphin into the BamHI/SacI site of pHAmi2638_SH3b2638. This construct was initially generated to allow for direct comparison with the His-tagged parental enzymes HPL2638 and HLST. All pQE-30-based plasmid constructs were transformed into E. coli XL1-Blue MRF′ (Agilent) by electroporation and verified by Sanger sequencing (GATC, Konstanz, Germany).

For further experiments with SA.100 (and its derivative XZ.700), non-His-tagged versions of these enzymes were obtained from Micreos Human Health (Bilthoven, The Netherlands). Compared to SA.100, XZ.700 features a deletion of a 44-amino-acid region at the N-terminal end of the Ply2638-derived protein fragment. Furthermore, two amino acids derived from the SacI restriction site originally used for cloning in the pQE-30-based constructs are no longer present in these non-His-tagged versions.

### Protein expression and purification.

Expression of recombinant proteins in E. coli was carried out essentially as previously described (33). In brief, bacterial cultures were grown under agitation in LB medium modified for protein expression (20) at 37°C and supplemented with suitable antibiotics for plasmid selection until an OD_600_ of 0.5 was reached. Following cooling on ice, 0.5 M isopropyl-β-d-thiogalactopyranoside (IPTG) was added for induction of protein expression, and incubation under agitation was continued for 18 h at 19°C. Cells were pelleted by centrifugation and frozen at −80°C. Depending on the presence of a His tag, pellets were thawed and resuspended in either lysis buffer for IMAC (50 mM NaH_2_PO_4_, 300 mM NaCl, 10 mM imidazole, and 30% glycerol, pH 8, for His-tagged proteins) or wash buffer for cation exchange chromatography (CIEX) (50 mM Na_2_HPO_4_, 50 mM NaCl, and 20% glycerol, pH 7.4, for proteins without His tag), and cells were disrupted by one passage through a Stansted Fluid Power pressure cell homogenizer at 100 MPa. His-tagged proteins were purified by IMAC as described before (20), using low-density nickel resin (ABT, Madrid, Spain), and eluted in IMAC elution buffer (50 mM NaH_2_PO_4_, 300 mM NaCl, 250 mM imidazole, and 30% glycerol, pH 8). Proteins without His tags were purified by CIEX as previously described (31), using a HiTrap Sepharose fast-flow (SP-FF) column on an Äkta fast-performance liquid chromatography (FPLC) device (GE Healthcare, Uppsala, Sweden), and eluted with a 1%/min gradient of CIEX elution buffer (50 mM Na_2_HPO_4_, 1 M NaCl, and 20% glycerol, pH 7.4). For *in vitro* experiments, proteins were dialyzed against phosphate-buffered saline with 0.01% Tween 20 (PBS-T). Dialyzed proteins were filter sterilized (0.2 μm), protein identity and purity evaluated by SDS-PAGE, and protein concentration measured with a NanoDrop ND-1000 spectrophotometer (NanoDrop Technologies, Wilmington, DE, USA). When proteins were produced for *in vivo* experiments, the purification procedure described above was modified to yield endotoxin-free preparations, essentially as previously described (20). Endotoxin concentrations were determined by using an EndoZyme kit (Hyglos, Regensburg, Germany) according to the manufacturer’s instructions. For experiments with reconstituted human epidermis and the murine skin infection model, XZ.700 was formulated as cetomacrogol-based oil-in-water cream (38). Additionally, a methylhydroxypropylcellulose-based gel formulation of XZ.700 was used for the *in vivo* model (15% methylhydroxypropylcellulose).

### TRAs.

TRAs were performed essentially as previously described (30). In brief, frozen S. aureus cells were thawed and diluted in PBS-T (pH 7.4), and aliquots of the suspension were mixed in a 96-well plate with endolysin dilutions in the same buffer, with final concentrations ranging from 12.5 to 200 nM, so that the initial OD_600_ of the suspensions was 1.0. Buffer without enzyme served as a negative control. The decrease in optical density over time in each well was monitored for 30 min at 10-s intervals using a FLUOstar Omega plate reader (BMG Labtech, Ortenberg, Germany). The resulting lysis curves were normalized, corrected for the no-enzyme control, and fitted to a sigmoidal function with 5 parameters as described before (73). The lytic activity of the endolysin under the conditions tested was calculated from the steepest slope of the fitted curve. To determine the temperature stability of endolysins, proteins at a concentration of 200 nM were heated to temperatures between 42°C and 72°C for 10 min, cooled down on ice, and then tested in a TRA as described above, in comparison with a nonheated control.

### qKAs.

To quantify the killing activity of endolysins, S. aureus was grown for 120 min in TSB to an OD_600_ of approximately 0.5 and harvested by centrifugation, and the pellet was resuspended in PBS-T to a concentration of 10^7^ CFU/mL. Aliquots (100 μL) of the suspension were mixed in 96-well plates with 100 μL each of serially diluted endolysins in PBS-T or buffer alone as a control. Plates were incubated at 37°C for 120 min. Then, 20-μL samples were removed from each well, serially diluted, and plated on TSB agar plates for enumeration of CFUs after overnight incubation.

### MIC assay.

MICs of SA.100 and XZ.700 against various staphylococcal strains were determined essentially as described before (74). In brief, 200 μL of an overnight culture of a staphylococcal strain was transferred to 5 mL TSB and incubated for 4 h at 35°C. Cells were spun down and resuspended in PBS to a density of 1 McFarland. This suspension was then diluted in cation-adjusted Mueller-Hinton broth (CAMHB) to a bacterial concentration of 10^5^ to 10^6^ CFU/mL. Stock solutions of endolysins were 2-fold serially diluted in CAMHB, and 25.6 μL of each enzyme dilution was mixed with 174.4 μL of bacterial suspension in a 96-well plate. After overnight incubation at 35°C, bacterial growth was assessed visually per well. The MIC was the lowest concentration at which no growth of the bacterium was observed.

### Mixed culture transwell experiments.

To determine the species-specific killing of S. aureus by XZ.700 in a mixed culture with S. epidermidis on a solid surface, both species were grown on 12-mm transwell cell culture inserts (Corning, Glendale, AZ, USA) with a 0.4-μm pore size. To this end, growth medium (1× M9 minimum salts [Sigma-Aldrich], 0.5 g/L casein amino acids, 0.5 g/L glucose, 2 mM MgSO_4_, 0.1 mM CaCl_2_, 1 mM thiamine, 0.05 mM nicotinamide, and 6 g/L agar) was filled into individual wells of a 12-well plate and allowed to solidify. Frozen stocks of S. aureus and S. epidermidis were diluted in minimal medium (1× M9 salts, 0.1 mM CaCl_2_, 1 mM thiamine, and 0.05 mM nicotinamide) (adapted from reference 75) to an OD_600_ of 0.1, and then, 50 μL of each strain was added to 9.9 mL medium. Two hundred microliters of this diluted mixed suspension were added to transwells (corresponding to approximately 10^4^ CFU/cm^2^) and centrifuged for 5 min at 2,000 × *g* to remove the liquid. The transwells were then inserted into agar-containing wells and incubated at 35°C in a humidity-controlled incubator. After 2 h of incubation, transwells were treated with 100 μg/mL XZ.700 in 100 μL PBS-T or buffer alone as a control (placebo). Treatment was stopped after 1 h by centrifugation to remove all liquid from the transwells, after which they were returned to the agar-containing wells and further incubated for 21 h at 35°C. At the end of the incubation period, bacteria were harvested from transwell membranes by filling the transwells with 500 μL PBS-T and gently pipetting up and down. This wash step was repeated with a further 500 μL PBS-T, and suspensions were pooled. The suspension was then serially diluted and plated onto CASO agar plates, CASO agar plates supplemented with 1 μg/mL fusidic acid, and CASO agar plates supplemented with 350 μg/mL potassium tellurite for enumeration of total bacteria, S. epidermidis only, and S. aureus only, respectively.

### Resistance induction assay.

Induction of resistance against XZ.700 and mupirocin was evaluated in two MSSA and two MRSA strains in duplicate by passing 20 consecutive cycles of exposure to subinhibitory concentrations (one-half MIC) of both agents and monitoring the MICs at every cycle. Every cycle consisted of a separate MIC determination as described above, with XZ.700 starting at a range of 32 μg/mL to 0.5 μg/mL in the first cycle and mupirocin at a range of 1,024 nM to 64 nM. Between every cycle, the cells in the wells at one-half the MIC (the well with the highest XZ.700 or mupirocin concentration still allowing visible growth) were transferred to 5 mL TSB, incubated for 4 h at 35°C, and harvested by centrifugation as described above. The pelleted cells served as inoculum for the next cycle of MICs. The fold change of MICs of XZ.700 and mupirocin compared to the respective MICs at the beginning of the experiment was plotted for each round of repeated exposure to visualize possible emergence of resistance. All strains used for this experiment were sensitive to mupirocin, as determined by the Vitek 2 system (bioMérieux, France) following the EUCAST standards (MICs ≤ 2 μg/mL).

### Activity of XZ.700 in an RHE model.

Reconstituted human epidermis (RHE; SkinEthic) samples for colonization with S. aureus and treatment with XZ.700 in solution and cream formulations were obtained from Episkin (Lyon, France). Colonization and treatment experiments were performed by VitroScreen (Milan, Italy). RHE samples (0.5 cm^2^) were cultivated for 17 days from normal human keratinocytes (Episkin) on inert 0.4-μm polycarbonate filters at the air-liquid interface in a chemically defined medium (Episkin), a procedure reproducing epidermal morphology. Each RHE batch was tested for the absence of HIV-1 and HIV-2, hepatitis B and hepatitis C viruses, and *Mycoplasma*. Immediately after arrival in the laboratory, the RHE samples were removed from the nutrient solution and rapidly transferred to 6-well plates previously filled with antibiotic-free maintenance medium (1 mL/well; Episkin) at room temperature and incubated overnight at 35°C, 5% CO_2_, and relative humidity of 90%.

An overnight culture of S. aureus strain ATCC 33591 in BHI broth was diluted to an OD_600_ of 0.1 and further incubated at 35°C for 2 h to reach the exponential-growth phase. Bacteria were then harvested by centrifugation and resuspended in a 0.9% saline solution at an OD_600_ of 0.1. The CFU/mL of this suspension was determined by serial dilution plating prior to using it for colonization of the RHE.

The maintenance medium of the RHE was exchanged, and 30 μL of S. aureus suspension was added, resulting in a bacterial concentration of approximately 5 × 10^6^ CFU/mL. Following incubation at 35°C for 4 h, all liquid was removed, and the colonized RHE was treated with 15 μL of XZ.700 solution at 32 μg/mL or 15 μL of XZ.700 cream at 3 different concentrations (32, 128, and 512 μg/mL) for either 30 min, 1 h, or 2 h. Untreated RHE served as a control. At the end of the treatment, 600 μL of medium was used to rinse the RHE surface by repeatedly pipetting up and down and subsequently collected to recover any nonadhered bacteria (apical fraction). The RHE tissue retaining any adhered bacteria was then collected in a tube containing 1.5 mL buffer solution and zirconium oxide beads and homogenized using a Minilys homogenizer (Bertin Corp., Rockville, MD, USA; 3 cycles of 30 s each at the lowest power). Bacterial concentrations in both apical fraction and tissue homogenate were determined by serial dilution plating on BHI agar.

### Mouse model of superficial skin infection.

Six- to 8-week-old female and male BALB/c mice (Charles River Laboratories, Wilmington, MA, USA) were housed in a pathogen-free environment on an *ad libitum* diet. All procedures performed on the mice were in accordance with the Animal Care and Use Committee of the Boston Children's Hospital.

The preparation of bacteria and infection of mice were carried out essentially as previously described (76). On the day of infection, an overnight culture of the bioluminescent MRSA strain USA300 LAC::*lux* was diluted 1:50 and incubated for another 2 h at 37°C. Bacteria were harvested and concentrated in PBS to approximately 2 × 10^9^ CFU/mL. The exact bacterial concentration was determined by serial dilution plating. Female or male mice were anesthetized with a mixture of ketamine and xylazine (60 to 100 mg/kg and 5 to 10 mg/kg body weight, respectively), and the back skin was shaved and tape stripped with Tegaderm 6 times. After 18 h, mice were anesthetized with a mixture of ketamine and xylazine and topically treated with 50 μL (≈1 × 10^8^ CFU) of the prepared S. aureus suspension on the tape-stripped skin with the help of a cotton swab.

In a preliminary study, the bacterial burden on female and male mice (*n* = 3 for each group) was determined over time. For this purpose, *in vivo* bioluminescence imaging was performed at different time points (8 h, 23 h, 31 h, 47 h, 56 h, 72 h, and 78 h) using a Pearl Trilogy small animal imaging system (LI-COR, Lincoln, NE, USA) (77). *In vivo* bioluminescence imaging data were presented on a color scale overlaid on a grayscale photograph of mice and quantified as total bioluminescence for a circular region (with background subtracted) using the Image Studio software (LI-COR). To enumerate the bacterial load on the skin at the end of the experiment, two 8-mm skin biopsy specimens were obtained. After mechanical homogenization using a Bio-Gen PRO200 homogenizer (Biogen, Cambridge, MA, USA) at ≈15,000 rpm for 1 min, serial dilutions of skin homogenates were cultured on CHROMagar plates.

For the treatment study, female mice were anesthetized and infected as described above in two independent experiments. Three hours postinfection, the infected skin was left untreated or treated with vehicle cream or gel (negative controls) or cream or gel containing XZ.700 at 30 μg/mL with the help of a cotton swab. The quantity of product applied onto the infected skin was calculated by the gravimetric difference between the cotton swab saturated with cream or gel before and after the treatment. Following the first treatment, further treatments were done twice per day at 9 a.m. at 5 p.m. for 3 days. The mice received 6 treatments in total. Before each treatment, the bacterial load on the infected skin was evaluated by *in vivo* bioluminescence imaging, and at the end of the experiment, S. aureus concentrations on the skin were determined, both as described above. Altogether, we conducted two separate experiments with 3 mice per group each (i.e., *n* = 6). The statistical power was 90%, with a significance level (α) of 0.05 (https://clincalc.com/).
