# Supplementary material for: Linker-Improved Chimeric Endolysin Selectively Kills Staphylococcus aureus In Vitro, on Reconstituted Human Epidermis, and in a Murine Model of Skin Infection
Source: Antimicrob Agents Chemother. 2022 Apr 13;66(5):e02273-21. doi: 10.1128/aac.02273-21 (PMC9112974; doi:10.1128/aac.02273-21)
Supplement: Supplemental file 1 — Fig. S1 to S3 and Table S1. Download aac.02273-21-s0001.pdf, PDF file, 1.0 MB [file aac.02273-21-s0001.pdf]

Supplementary Material

A

MAATHEHSAQWLNNYKKGYGYGPYPLGINGGMHYGVDFFMNIGTPVKAISSGKIVEAGWSNYGGGNQIGLIENDGV
HRQWYMHLSKYNVKVGDYVKAGQIIGWSGSTGYSTAPHLHFQRMVNSFSNSTAQDPMPFLKSAGYGKAGGTVTPT
PNTGELLRPKDAKKDEKSQVCSGLAMEKYDITNLNAKQDKSKNGSVKELKHIYSNHIKGNKITAPKPSIQGVVIHNDYGS
MTPSQYLPWLYARENNGTHVNGWASVYANRNEVLWYHPTDYVEWHCGNQWANANLIGFEVCESYPGRISDKLFLE
NEEATLKVAADVMKSYGLPVNRNTVRLHNEFFGTSCPHRSWDLHVGKGEPYTTTNINKMKDYFIKRIKHYYDGGKLEV
SKAATIKQSDVKQEVKKQEAKQIVKATDWKQNKDGIWYKAEHASFTVTAPEGIITRYKGPWTGHPQAGVLQKGQTIKY
DEVQKFDGHVWVSWETFEGETVYMPVRTWDAKTGKVGKLWGEIK

B

MAATHEHSAQWLNNYKKGYGYGPYPLGINGGMHYGVDFFMNIGTPVKAISSGKIVEAGWSNYGGGNQIGLIENDGV
HRQWYMHLSKYNVKVGDYVKAGQIIGWSGSTGYSTAPHLHFQRMVNSFSNSTAQDPMPFLKSAGYGKAGGTVTPT
PNTGLKHIYSNHIKGNKITAPKPSIQGVVIHNDYGSMTPSQYLPWLYARENNGTHVNGWASVYANRNEVLWYHPTDY
VEWHCGNQWANANLIGFEVCESYPGRISDKLFLENEEATLKVAADVMKSYGLPVNRNTVRLHNEFFGTSCPHRSWDL
HVGKGEPYTTTNINKMKDYFIKRIKHYYDGGKLEVSKAATIKQSDVKQEVKKQEAKQIVKATDWKQNKDGIWYKAEHA
SFTVTAPEGIITRYKGPWTGHPQAGVLQKGQTIKYDEVQKFDGHVWVSWETFEGETVYMPVRTWDAKTGKVGKLW
GEIK

Supplementary Figure 1:

Amino acid sequences of the chimeric enzymes SA.100 (**A**) and XZ.700 (**B**). The 44 amino acid region that has been deleted in XZ.700 as compared to SA.100 is underlined in panel A. Lysostaphin-derived sequence portions are displayed in green, whereas those originating from staphylococcal phage endolysin Ply2638 are shown in orange/gold. The conserved M23 domain of lysostaphin, the amidase domain of Ply2638, and the SH3b domain of Ply2638 are displayed in dark green, gold, and dark gold, respectively. The amino acids "EL" shown in red in panel A are derived from the SacI restriction site that had been used during cloning for generation of SA.100.

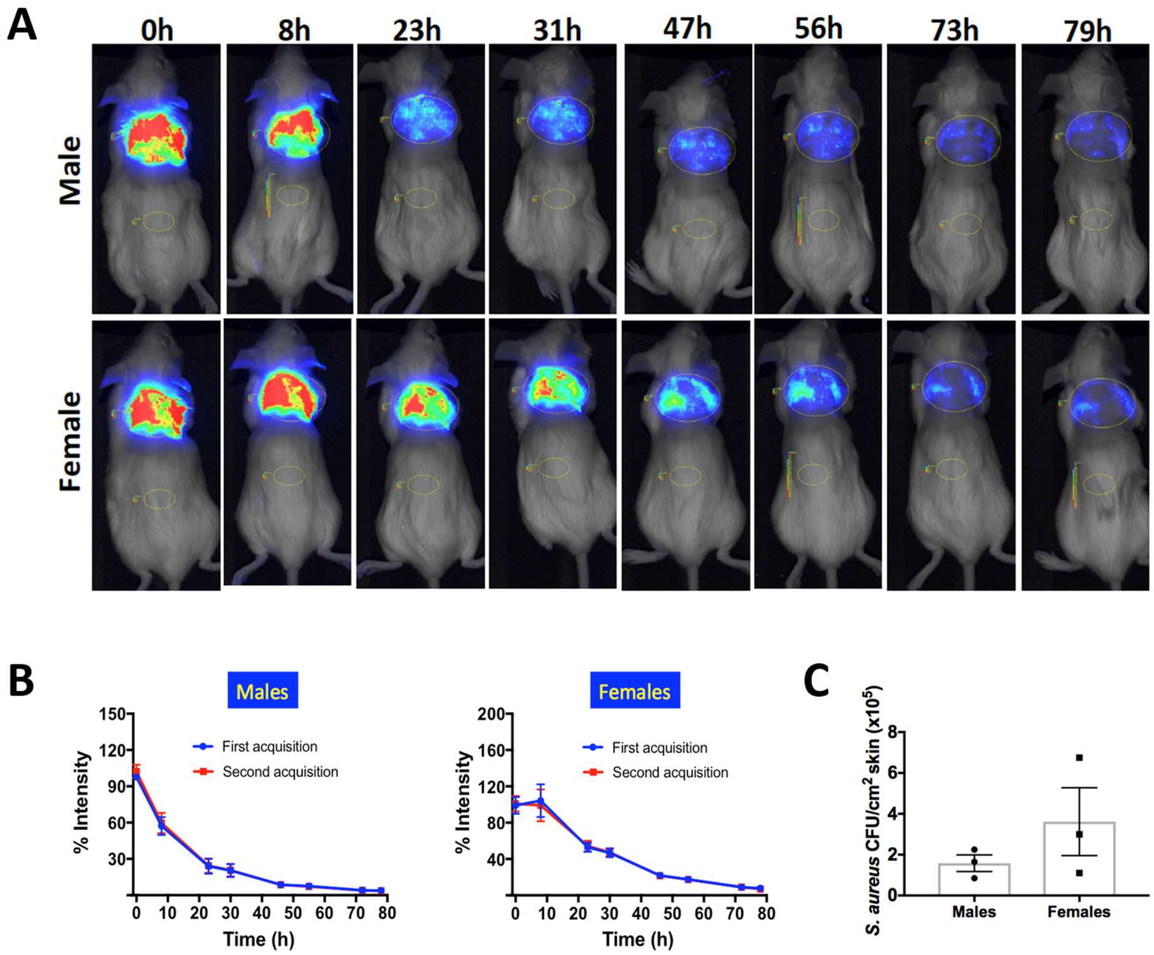

Supplementary Figure 2:

Clearance of *S. aureus* over time from the skin of tape-stripped male and female mice. **(A)** Representative bioluminescence images illustrating the concentration of *S. aureus* on male and female mice immediately after infection (t = 0h) and for up to 79 h. **(B)** Quantification of luminescence intensity over time obtained from images as exemplified in panel A (n = 3). **(C)** *S. aureus* CFU counts/cm^2 on male and female mice at 79 h after infection (n = 3).

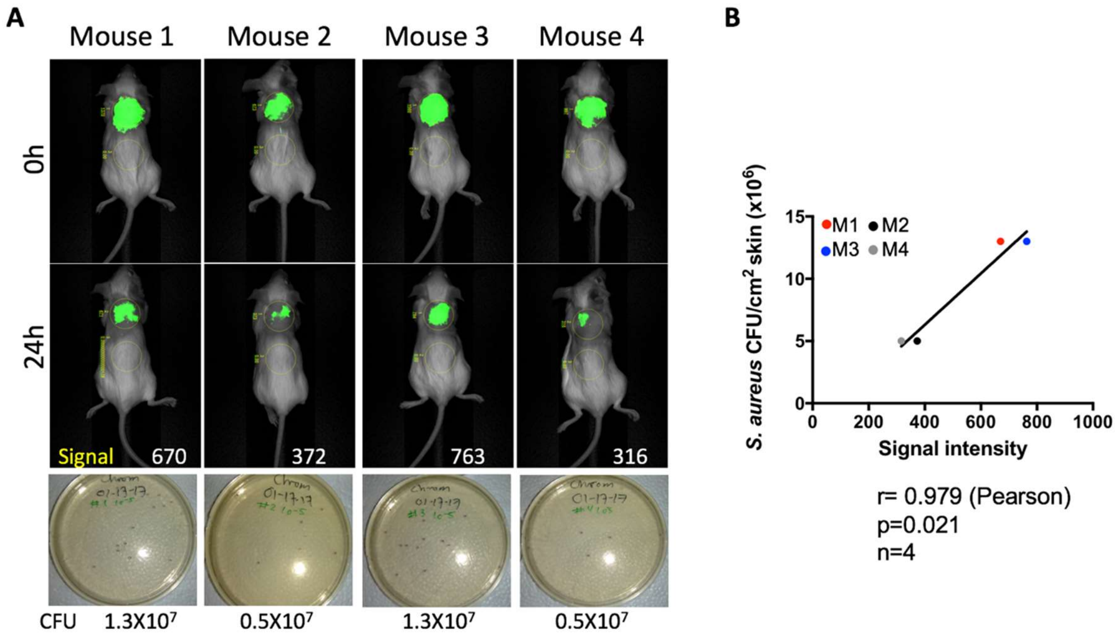

Supplementary Figure 3:

Correlation between bioluminescence signal intensity and CFU counts from skin homogenates 24 h after superficial *S. aureus* infection. **A.** Representative bioluminescence images of tape-stripped mice infected with the bioluminescent *S. aureus* strain USA300 LAC::*lux* immediately (0h) and 24 h after superficial infection (24h) and CFU counts in homogenates obtained at 24 h. **B**. Correlation plot between bioluminescence signal intensity and CFU counts from skin homogenates at 24 h.

Supplementary Table 1: Minimum inhibitory concentrations (MICs) of XZ.700 against multiple *Staphylococcus aureus* (MSSA and MRSA) and non-*S. aureus* (control) strains from different geographic regions (n = 1)

No.	Isolate ID	Category	Species	Origin	Country	Remarks	XZ.700 MIC (μg/ml)
1	161227024501-1	MSSA	*Staphylococcus aureus*	blood	The Netherlands		2
2	161227017901-1	MSSA	*Staphylococcus aureus*	blood	The Netherlands		4
3	170201053701-1	MSSA	*Staphylococcus aureus*	blood	The Netherlands	Fucidic acid R	2
4	170303020801-1	MSSA	*Staphylococcus aureus*	blood	The Netherlands	Fucidic acid R	2
5	170601022701-1	MSSA	*Staphylococcus aureus*	blood	The Netherlands	Fucidic acid R	4
6	170609021401-1	MSSA	*Staphylococcus aureus*	blood	The Netherlands	Fucidic acid R	2
7	170814016001-1	MSSA	*Staphylococcus aureus*	blood	The Netherlands		8
8	170529015901-1	MSSA	*Staphylococcus aureus*	blood	The Netherlands		8
9	170527001701-1	MSSA	*Staphylococcus aureus*	blood	The Netherlands		2
10	170511026901-1	MSSA	*Staphylococcus aureus*	blood	The Netherlands		0.5
11	170526061901-1	MSSA	*Staphylococcus aureus*	blood	The Netherlands		4
12	170102033901-1	MSSA	*Staphylococcus aureus*	burn wound	The Netherlands		8
13	161230032701-3	MSSA	*Staphylococcus aureus*	burn wound	The Netherlands	Fucidic acid R	8
14	161229022501-3	MSSA	*Staphylococcus aureus*	burn wound	The Netherlands	Fucidic acid R	4
15	170105033301-1	MSSA	*Staphylococcus aureus*	burn wound	The Netherlands	Fucidic acid R	4
16	170223065201-1	MSSA	*Staphylococcus aureus*	burn wound	The Netherlands	Fucidic acid R	2
17	170619040001-1	MSSA	*Staphylococcus aureus*	burn wound	The Netherlands	Fucidic acid R	2
18	170220026901-1	MSSA	*Staphylococcus aureus*	burn wound	The Netherlands	Fucidic acid R	2
19	170406066601-3	MSSA	*Staphylococcus aureus*	burn wound	The Netherlands	Fucidic acid R	2
20	170526034401-1	MSSA	*Staphylococcus aureus*	burn wound	The Netherlands	Fucidic acid R	2
21	170907064201-2	MSSA	*Staphylococcus aureus*	burn wound	The Netherlands	Fucidic acid R	2
22	170814035601-1	MSSA	*Staphylococcus aureus*	burn wound	The Netherlands		8
23	170815032601-1	MSSA	*Staphylococcus aureus*	burn wound	The Netherlands		4
24	170720049601-1	MSSA	*Staphylococcus aureus*	burn wound	The Netherlands		2
25	170608049201-1	MSSA	*Staphylococcus aureus*	burn wound	The Netherlands		2
26	170426041401-1	MSSA	*Staphylococcus aureus*	burn wound	The Netherlands		2
27	170810033801-1	MSSA	*Staphylococcus aureus*	joint aspirate	The Netherlands		2
28	161229029801-1	MSSA	*Staphylococcus aureus*	joint aspirate	The Netherlands		4

29	171025047001-1	MSSA	*Staphylococcus aureus*	nose	The Netherlands		1
30	171025046701-1	MSSA	*Staphylococcus aureus*	nose	The Netherlands		8
31	171025046301-1	MSSA	*Staphylococcus aureus*	nose	The Netherlands		4
32	171025046101-1	MSSA	*Staphylococcus aureus*	nose	The Netherlands		4
33	171025046001-1	MSSA	*Staphylococcus aureus*	nose	The Netherlands		2
34	171025045901-1	MSSA	*Staphylococcus aureus*	nose	The Netherlands		4
35	171026037301-1	MSSA	*Staphylococcus aureus*	nose	The Netherlands	Fucidic acid R	4
36	170201064901-1	MSSA	*Staphylococcus aureus*	pleural empyema	The Netherlands		8
37	AZP17-17350	MSSA	*Staphylococcus aureus*	pus	Surinam		4
38	170823027001-1	MSSA	*Staphylococcus aureus*	pus	The Netherlands		8
39	171003056101-1	MSSA	*Staphylococcus aureus*	skin	The Netherlands	Fucidic acid R	2
40	150909037501-1	MSSA	*Staphylococcus aureus*	skin	The Netherlands	Fucidic acid I	2
41	150911021401-1	MSSA	*Staphylococcus aureus*	skin	The Netherlands	Fucidic acid I	1
42	151010019801-1	MSSA	*Staphylococcus aureus*	skin	The Netherlands	Fucidic acid R	2
43	160505000401-1	MSSA	*Staphylococcus aureus*	skin	The Netherlands		4
44	17072040301-1	MSSA	*Staphylococcus aureus*	sputum	The Netherlands	Mupirocin R	2
45	170424029401-1	MSSA	*Staphylococcus aureus*	sputum	The Netherlands		2
46	170606037501-1	MSSA	*Staphylococcus aureus*	tissue	The Netherlands		2
47	AZP17-17255	MSSA	*Staphylococcus aureus*	wound	Surinam		8
48	AZP17-17343	MSSA	*Staphylococcus aureus*	wound	Surinam		4
49	151030042101-1	MSSA	*Staphylococcus aureus*	wound	The Netherlands		16
50	170202069101-1	MSSA	*Staphylococcus aureus*	wound	The Netherlands	Fucidic acid R	4
51	150617038301	MRSA	*Staphylococcus aureus*	abscess	Nepal		8
52	161230032901	MRSA	*Staphylococcus aureus*	abscess	Philippines		4
53	AZP17-2341	MRSA	*Staphylococcus aureus*	blood	Surinam		2
54	AZP17-4053	MRSA	*Staphylococcus aureus*	blood	Surinam		2
55	AZP17-9084	MRSA	*Staphylococcus aureus*	blood	Surinam		4
56	AZP17-12179	MRSA	*Staphylococcus aureus*	CVC	Surinam		4
57	160803033901	MRSA	*Staphylococcus aureus*	nose	Australia		2
58	160629065801	MRSA	*Staphylococcus aureus*	nose	Germany		8
59	160908063801	MRSA	*Staphylococcus aureus*	nose	Greece		4
60	160915040201	MRSA	*Staphylococcus aureus*	nose	Malaysia		16
61	150324048101	MRSA	*Staphylococcus aureus*	nose	Spain		2
62	160106044001	MRSA	*Staphylococcus aureus*	nose	Syria		2
63	170217039601	MRSA	*Staphylococcus aureus*	nose	Syria		4

64	170502030101	MRSA	*Staphylococcus aureus*	nose	Syria	4
65	161108035601	MRSA	*Staphylococcus aureus*	nose	Turkey	2
66	161108082901	MRSA	*Staphylococcus aureus*	nose	Turkey	2
67	170404051901	MRSA	*Staphylococcus aureus*	perineum	Afghanistan	4
68	160803058501	MRSA	*Staphylococcus aureus*	perineum	Germany	2
69	160924029101	MRSA	*Staphylococcus aureus*	perineum	Hungary	2
70	170303041501	MRSA	*Staphylococcus aureus*	perineum	Syria	4
71	150430043601	MRSA	*Staphylococcus aureus*	pus	Thailand	2
72	160607046301	MRSA	*Staphylococcus aureus*	skin	Aruba	2
73	151027058901	MRSA	*Staphylococcus aureus*	skin	Czech Republic	8
74	150212029501	MRSA	*Staphylococcus aureus*	skin	India	4
75	151103048201	MRSA	*Staphylococcus aureus*	skin	Syria	4
76	160425057101	MRSA	*Staphylococcus aureus*	skin	Turkey	8
77	150430030901	MRSA	*Staphylococcus aureus*	throat	China	8
78	160802055501	MRSA	*Staphylococcus aureus*	throat	Eritrea	1
79	150522023601	MRSA	*Staphylococcus aureus*	throat	France	4
80	170329039701	MRSA	*Staphylococcus aureus*	throat	Iraq	4
81	160622066901	MRSA	*Staphylococcus aureus*	throat	Italy	4
82	160605006101	MRSA	*Staphylococcus aureus*	throat	South-Africa	16
83	151026035501	MRSA	*Staphylococcus aureus*	throat	Spain	2
84	AZP17-10788	MRSA	*Staphylococcus aureus*	throat	Surinam	2
85	160517038601	MRSA	*Staphylococcus aureus*	throat	Syria	4
86	160115043001	MRSA	*Staphylococcus aureus*	throat	Turkey	2
87	170307028901	MRSA	*Staphylococcus aureus*	throat	Turkey	4
88	161112028101	MRSA	*Staphylococcus aureus*	vagina	Egypt	16
89	151015030201	MRSA	*Staphylococcus aureus*	wound	Curacao	4
90	160909069801	MRSA	*Staphylococcus aureus*	wound	Germany	8
91	150409053401	MRSA	*Staphylococcus aureus*	wound	Indonesia (Bali)	8
92	150910021801	MRSA	*Staphylococcus aureus*	wound	Morocco	16
93	160920041601	MRSA	*Staphylococcus aureus*	wound	Papua-NG	8
94	150826040101	MRSA	*Staphylococcus aureus*	wound	Philippines	2
95	170124065101	MRSA	*Staphylococcus aureus*	wound	Spain	4
96	AZP17-11421	MRSA	*Staphylococcus aureus*	wound	Surinam	2
97	AZP17-5000	MRSA	*Staphylococcus aureus*	wound	Surinam	4
98	160827011901	MRSA	*Staphylococcus aureus*	wound	Vietnam	4

99	ATCCBAA-1717	MRSA	*Staphylococcus aureus*	PFGE: USA300	ATCCBAA-1717	4
100	ATCCBAA-1707	MRSA	*Staphylococcus aureus*	PFGE: USA400	ATCCBAA-1707	4
101	160316027101-1	Control	*Corynebacterium jeikeium*	blood	The Netherlands	>256
102	160312011601-1	Control	*Cutibacterium acnes*	blood/skin*	The Netherlands	>256
103	160222049101-1	Control	*Saccharomyces cerevisiae*	quality control	The Netherlands	>256
104	170814016001-2	Control	*Staphylococcus capitis*	blood	The Netherlands	>256
105	160314033401-1	Control	*Staphylococcus capitis*	skin	The Netherlands	>256
106	151230040701-1	Control	*Staphylococcus chromogenes*	mastitis (cow)**	The Netherlands	<2
107	161111065901-1	Control	*Staphylococcus cohnii*	quality control	The Netherlands	>256
108	170829020401-1	Control	*Staphylococcus epidermidis*	blood	The Netherlands	>256
109	170820000801-1	Control	*Staphylococcus epidermidis*	blood	The Netherlands	>256
110	170810016301-1	Control	*Staphylococcus epidermidis*	liquor	The Netherlands	>256
111	170813003801-1	Control	*Staphylococcus hominis*	blood	The Netherlands	>256
112	170815015301-1	Control	*Staphylococcus hominis*	blood	The Netherlands	>256
113	170810020301-1	Control	*Staphylococcus hominis*	blood	The Netherlands	>256
114	170113059301-1	Control	*Staphylococcus lugdunensis*	joint aspirate	The Netherlands	8
115	170824024501	Control	*Staphylococcus lugdunensis*	tissue	The Netherlands	8
116	170510045101-1	Control	*Staphylococcus lugdunensis*	urine	The Netherlands	8
117	140425015259-3	Control	*Staphylococcus pseudintermedius*	wound (dog)	The Netherlands	<2
118	140425015271-4	Control	*Staphylococcus pseudintermedius*	wound (dog)	The Netherlands	<2
119	140528015136-2	Control	*Staphylococcus pseudintermedius*	wound (dog)	The Netherlands	<2
120	170627038701-1	Control	*Staphylococcus warneri*	quality control	The Netherlands	>256

* Skin contaminant in blood culture bottle.

** Culture of udder of cow with mastitis.
